# Nature-Derived Cellulose-Based Composite Separator for Sodium-Ion Batteries

**DOI:** 10.3389/fchem.2020.00153

**Published:** 2020-03-10

**Authors:** Jae Hyeon Jo, Chang-Heum Jo, Zhengfu Qiu, Hitoshi Yashiro, Liyi Shi, Zhuyi Wang, Shuai Yuan, Seung-Taek Myung

**Affiliations:** ^1^Department of Nanotechnology and Advanced Materials Engineering & Sejong Battery Institute, Sejong University, Seoul, South Korea; ^2^Research Centre of Nanoscience and Nanotechnology, Shanghai University, Shanghai, China; ^3^Department of Chemistry and Bioengineering, Iwate University, Iwate, Japan

**Keywords:** cellulose, composite, separator, sodium, battery

## Abstract

Sodium-ion batteries (SIBs) are emerging power sources for the replacement of lithium-ion batteries. Recent studies have focused on the development of electrodes and electrolytes, with thick glass fiber separators (~380 μm) generally adopted. In this work, we introduce a new thin (~50 μm) cellulose–polyacrylonitrile–alumina composite as a separator for SIBs. The separator exhibits excellent thermal stability with no shrinkage up to 300°C and electrolyte uptake with a contact angle of 0°. The sodium ion transference number, $t_{\text{Na}}^{+}$, of the separator is measured to be 0.78, which is higher than that of bare cellulose ($t_{\text{Na}}^{+}$: 0.31). These outstanding physical properties of the separator enable the long-term operation of NaCrO_2_ cathode/hard carbon anode full cells in a conventional carbonate electrolyte, with capacity retention of 82% for 500 cycles. Time-of-flight secondary-ion mass spectroscopy analysis reveals the additional role of the Al_2_O_3_ coating, which is transformed into AlF_3_ upon long-term cycling owing to HF scavenging. Our findings will open the door to the use of cellulose-based functional separators for high-performance SIBs.

## Introduction

**Scheme 1:**
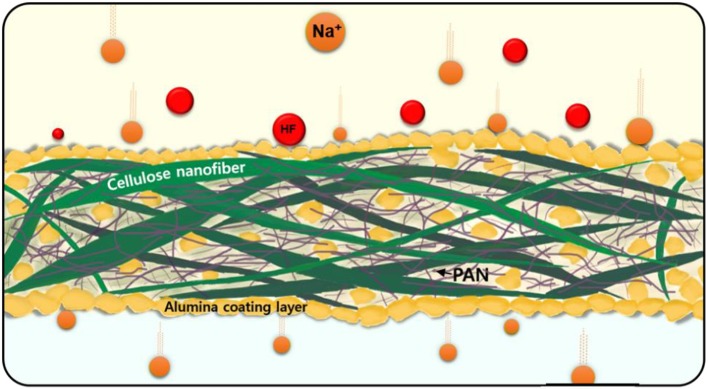
Schematic illustration of a cellulose-polyacrlonitrile-alumina separator.

Lithium-ion batteries (LIBs) have received significant attention worldwide as major energy sources for electric vehicles and mobile devices, and the demand for LIBs is expected to increase in the near future (Mizushima et al., 1980; Amatucci et al., 1996; Winter et al., 1998; Mishra and Ceder, 1999; Sun et al., 2009; Jo et al., 2015). However, the cost increase of lithium resources resulting from their limited supply will make satisfying this demand difficult; thus, the development of post-LIB battery systems is essential (Pan et al., 2013; Nithya and Gopukumar, 2015; Park et al., 2017; Jo et al., 2018a,b; Jo J. H. et al., 2018; Vaalma et al., 2018). Recently, sodium-ion batteries (SIBs) have been highlighted as attractive alternatives to LIBs for energy storage for both economic and scientific reasons; namely, sodium reserves are abundant and the reaction chemistries adopting a monovalent charge carrier are similar (Kim et al., 2015; Choi et al., 2018; Konarov et al., 2018). However, one shortcoming of SIBs is the slightly higher standard electrode potential of sodium [−2.7 V vs. the standard hydrogen electrode (SHE)] relative to that of lithium (−3.04 V vs. SHE), which has motivated the development of high-energy-density cathode materials and high-capacity anode materials (Yabuuchi et al., 2012, 2013; Guignard et al., 2013; Vassilaras et al., 2013). There have been many reports on high-energy-density cathode materials with surface modification to prevent particle damage from the changing volume during charge/discharge, anode materials with high reversible capacity, and stable electrolytes under oxidizing environments (Yu et al., 2015; Hwang et al., 2017; Åvall et al., 2018; Kim et al., 2018; Sato et al., 2018; Suharto et al., 2018; Choi et al., 2019; Jo et al., 2019; Lee et al., 2019; Wang et al., 2020). In most of these studies, glass fibers (GF) have been widely used in most SIBs because of their advantages over excellent wettability for ethylene carbonate and propylene carbonate, indicating high porosity (66%), large electrolyte uptake (360%), high ionic conductivity of electrolyte soaked in separator (3.8 mS cm^−1^), sodium ion transfer number ($t_{\text{Na}}^{+}$ = 0.79) than polypropylene membrane (Zhu et al., 2016; Arunkumar et al., 2019). Apart from the acceptable physical properties, cost and thickness of GF separator may impede their adoption to commercial SIBs at present. Therefore, finding an alternative to GF separators is urgent for commercialization of SIBs (Suharto et al., 2018). For these reasons, there have been few studies using polyolefin membranes modified by oxides or polymers (Kim et al., 2001, 2004, 2009; Jeng and Kim, 2004). Separators must provide adequate (1) porosity, (2) wettability, (3) chemical and thermal stability, and (4) ionic conductivity of electrolyte soaked in separator. Polypropylene (PP) and polyethylene (PE) separators are not suitable as a separator for batteries, because the separators exhibit very low wettability for cyclic carbonate-based electrolytes such as ethylene carbonate (EC) and propylene carbonate (PC), which are used as electrolytes for SIBs (Suharto et al., 2018). Their low thermal property is considered as another difficulty to apply them for SIBs (Xu, 2004; Kim et al., 2014; Zhu et al., 2016). The advantages of cellulose-based membranes for LIBs include their electrolyte uptake, thermal stability, and high ionic conductivity of electrolyte soaked in separator, which lead to high rate capability (Chiapponea et al., 2011). Chen et al. reported the suitability of cellulose acetate separators in SIBs for long-term cycling (Chen et al., 2018). Herein, we propose a new cellulose–polyacrylonitrile–alumina composite as a SIB separator (Scheme 1). Cellulose nanofibers are easily obtained from plants and are composed of close-packed polysaccharide chains with β- (1 → 4) -D-glucopyranose repeating units (Siró and Plackett, 2010). Cellulose nanofibers provide advantages in the process of separating membranes owing to their excellent dispersing properties in various solvents and inherent porous nanostructure, good thermal stability (melting point ≥ 250°C), hydrophilicity, and chemical stability (Chun et al., 2011). However, the cellulose membrane, which is thinner than that of a glass fiber, can be vulnerable to dendrite growth of sodium metal; thus, the mechanical strength must be improved for the application of cellulose as a separator. To overcome this issue, a commercial cellulose–PAN membrane was compositized with Al_2_O_3_ particles by dip coating, where the addition of Al_2_O_3_ was anticipated to provide thermal stability and functionality during the electrochemical reaction with the electrolyte. In addition, PAN is known to improve not only the mechanical properties but also the ionic conductivity of electrolyte soaked in separator. As designated, the present cellulose–PAN–Al_2_O_3_ composite separator exhibits acceptable physico-chemical properties, namely, a Gurley value of 153.3 s, ionic conductivity of electrolyte soaked in separator of 0.751 mS cm^−1^, transference number of sodium ions of 0.78, contact angle of 0°, puncture strength of 2.22 MPa, and thermal stability up to 300°C without shrinkage. The applicability of the cellulose–PAN–Al_2_O_3_ composite separator was verified in full cells fabricated with a NaCrO_2_ cathode // hard carbon anode in a conventional carbonate electrolyte for long-term cycling, resulting in capacity retention of 82% at a rate of 10 C for 500 cycles. Our findings suggest that the cellulose–PAN–Al_2_O_3_ composite separator is eminently suitable for use in SIBs.

## Experimental Section

### Preparation of Cellulose-PAN-Al_2_O_3_ Composite Separator

Al_2_O_3_ coating solution was provided by the Emerging Industries Institute of Shanghai University. A cellulose/PAN hybrid (cellulose/PAN = 50/50 in weight) electrospun nano-fiber separator (thickness: 21 μm) was obtained from NingBo EneRol Nanotechnology, Inc. For dip coating of the separator, the pristine cellulose–PAN separator was immersed in an Al_2_O_3_ suspension, composed of polyvinyl acetate (PVAc) as a binder and aqueous Al_2_O_3_ dispersion, provided by the Emerging Industries Institute of Shanghai University. The coated separators were dried at 60°C for 0.5 h. The composited separators were thickened to 50 μm by repeated dip-coating and drying processes. The composited cellulose–PAN–Al_2_O_3_ separators were further vacuum-dried at 100°C for 12 h prior to use.

### Characterization

X-ray diffraction (XRD; X'Pert, PANalytical) was employed to identify the phases of the coated materials on the cellulose–PAN separator. XRD data collection was performed in the 2θ range of 10-110°. Field-emission scanning electron microscopy (FE-SEM; Hitachi S-4700) was used to examine the morphology and cross-section of the composite separator. The functionality of the coated Al_2_O_3_ on the cellulose–PAN separator was identified using time-of-flight secondary ion mass spectroscopy (ToF-SIMS; PHI TRIFT V nanoTOF, ULVAC-PHI). The tensile strength of the separator was determined using a JDL-200N tensile tester with a tensile speed of 100 mm min^−1^.

Contact angle measurements of the cellulose–PAN and cellulose–PAN–Al_2_O_3_ composite separators were performed using an OCA 15EC optical contact angle measuring device (Data Physics Instruments, Germany) at room temperature using the sessile drop method. The air permeability of the separators was evaluated using a Gurley densometer (UEC, 1012A) that measures the time required for 100 cc of air to pass through the separator. The electrolyte uptake of the separator was calculated using the following equation:

$$\text{Electrolyte Uptake }(\text{wt\%})=\frac{\text{w}-{\text{w}}_{0}}{{\text{w}}_{0}}\times 100\%,$$

where W_0_ is the weight of the dry separator and W is the total weight of the separator and soaked liquid electrolyte.

The ionic conductivity (σ) of electrolyte soaked in separator was measured by analyzing the AC impedance spectra of stainless steel (SS)/separator/SS cells on an AutoLab (PGSTAT302N, Metrohm) electrochemical workstation in the range of 0.1 Hz−1 MHz at room temperature using the following equation:

$$\sigma \text{=}\frac{\text{L}}{{\text{R}}_{\text{b}}.\text{S}}.$$

Here, L is the thickness of the separator, S is the contact area of the separator and stainless-steel electrode, and R_b_ is the bulk resistance.

The puncture strength of the separator was determined using a Labthink Instrument XLW-PC auto tensile tester at a testing speed of 100 mm min^−1^.

The Na^+^ transference number (${\text{t}}_{{\text{Na}}^{\text{+}}}$) was measured using a combination of chronoamperometry and electrochemical impedance spectroscopy (EIS) of the sodium metal/separator–electrolyte/sodium metal cell using the following equation:

$${\text{t}}_{{\text{Na}}^{\text{+}}}\text{=}\frac{{\text{I}}_{\text{s}}(\Delta {\text{V}-\text{I}}_{\text{0}}{\text{R}}_{0})}{{\text{I}}_{0}(\Delta {\text{V}-\text{I}}_{\text{s}}{\text{R}}_{\text{s}})}.$$

Here, ΔV is the potential difference set as 10 mV; I_0_ is the initial current obtained; I_s_ is the steady-state current; and R_0_ and R_s_ are the initial and steady-state interfacial resistance after polarization, respectively.

The thermal shrinkage ratio (TSR) was used to evaluate the thermal stability of the separators:

$$\text{TRS(\%)}=\frac{{\text{S}}_{0}-\text{S}}{{\text{S}}_{0}}\times 100\%,$$

where S_0_ and S are the area of the separator before and after thermal treatment for 0.5 h at different temperatures, respectively. Thermogravimetric analysis (TGA) and differential scanning calorimetry (DSC) were performed by heating from 30 to 800°C in a nitrogen (N_2_) atmosphere using a Netzsch simultaneous thermal analyzer (STA 449 F1 Jupiter).

### Electrochemical Characterization

To fabricate a cathode, carbon-coated NaCrO_2_ (C-NaCrO_2_) was blended with conducting materials (a mixture of Super P carbon black and Ketjen black in a 1:1 ratio) and polyvinylidene fluoride (PVDF) in an 8:1:1 weight ratio in *N*-methyl pyrrolidone (NMP) solution. The obtained slurry was applied on Al foil, dried at 80°C for several hours to remove the NMP, and then heated at 120°C overnight under vacuum. Electrochemical cell tests were performed after assembling R2032 coin-type cells using Na metal or hard carbon as the negative electrode separated by the cellulose–PAN–Al_2_O_3_ composite separator. The assembly was performed in an argon-filled glove box. The electrolyte solution was 0.5 mol dm^−3^ NaPF_6_ in ethylene carbonate (EC):dimethyl carbonate (DMC) (1:1 by volume), in which fluoroethylene carbonate (FEC) was added at a ratio of 3 vol.% vs. the EC:DMC solvent (Komaba et al., 2011). For full cell preparation, hard carbon anodes were pre-sodiated via electrochemical cycling of the anode in the range of 0.01–2 V, to minimize the first irreversible capacity of cathode and form solid electrolytic interphase on the hard carbon anode. Before cycling for hard carbon anode, it first was pre-potassiated through direct contact with potassium metal in 0.5 mol dm^−3^ NaPF_6_ electrolyte for 1 h to minimize the irreversibility during sodiation process. Loading levels of active material were 3.0 mg cm^−2^ for the cathode and 1.3 mg cm^−2^ for the anode, which approaches to a N/P ratio of 1.2.

## Results and Discussion

**Figure 1:**
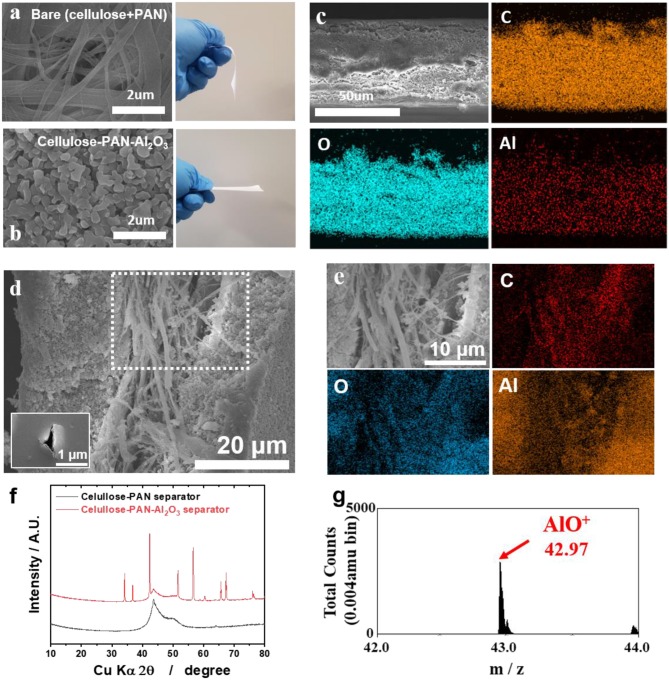
**(a)** SEM image and photograph of cellulose–PAN separator; **(b)** SEM image of cellulose–PAN–Al_2_O_3_ composite separator; **(c)** SEM image and EDX mapping data (Al, C, O elements) of cellulose–PAN-Al_2_O_3_ composite separator; **(d)** SEM image on side for cellulose–PAN-Al_2_O_3_ composite separator (inset: SEM image of cellulose–PAN-Al_2_O_3_ composite separator at pierced point). **(e)** The magnified SEM image of cellulose–PAN-Al_2_O_3_ composite separator with EDX mapping result for Al, C, and O elements. **(f)** XRD pattern of bare and cellulose–PAN-Al_2_O_3_ composite separator; **(g)** ToF-SIMS data of cellulose–PAN-Al_2_O_3_ composite separator for AlO^+^ (m = 42.97) fragment.

The cellulose–PAN separator was fabricated to achieve randomly arranged smooth fibers by electrospinning a homogeneous solution of a 1:1 mixture of cellulose and PAN, forming a non-woven separator. The cellulose itself was floppy with large pores; fabricating composite with PAN was expected to provide mechanical strength to the separator. In addition, the reinforced mechanical strength enabled the shape of the separator to be maintained, preventing the generation of an electrical short-circuit between the cathode and anode to enable facile diffusion of charge carriers (Figure 1a). Insulating and thermally stable Al_2_O_3_ powders with submicron size (<500 nm) were coated onto the cellulose–PAN separator to improve the thermal stability and ensure the porosity of the separator, namely, fabrication of a cellulose–PAN–Al_2_O_3_ composite separator with a thickness of 50 μm (Figure 1c) and EDX mapping image presented the presence of Al_2_O_3_ together with the cellulose–PAN separator (Figure 1c). The cellulose-PAN-Al_2_O_3_ separator was punctured by a needle to check the laminated structure of the separator (Figures 1d,e). The separator is composed of cellulose (> 1 μm thickness fiber), polyacrylonitrile (PAN, < 1 μm thickness fiber), and Al_2_O_3_, in which both fibers are non-woven together but presented together (Figure 1d). Therefore, it is thought that the presence of tensile PAN is likely to improve the mechanical strength of the cellulose in the cellulose-PAN separator. Based on SEM with EDX image, the side of separator presented clear distribution of Al, C, and O elements as shown in Figures 1d,e. In other words, it demonstrated that the cellulose, PAN, and Al_2_O_3_ coexist uniformly in the separator, as shown in Scheme 1. Furthermore, XRD pattern confirmed the presence of Al_2_O_3_ together with the cellulose–PAN separator (Figure 1f), and ToF-SIMS analysis provided further information on the presence of Al–O chemical bonding, which was attributed to the AlO^+^ fragment (m = 42.97) (Figure 1g).

**Figure 2:**
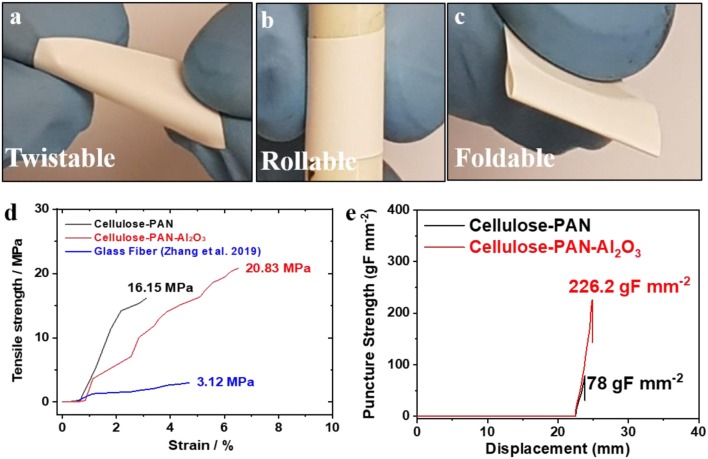
**(a)** Twisting, **(b)** rolling, and **(c)** folding test results for cellulose–PAN-Al_2_O_3_ composite separator; **(d)** stress–strain curves of uncoated (bare) and cellulose–PAN-Al_2_O_3_ composite separator; **(e)** puncture strength curves of uncoated (bare) and cellulose–PAN-Al_2_O_3_ composite separator.

Separators must have intimate contact with both the cathode and anode; thus, good flexibility, and tensile strength are important prerequisites for separators in batteries. As shown in Figures 2a–c, the as-prepared cellulose–PAN–Al_2_O_3_ composite separators could be twisted, rolled, and folded without damage under various bending conditions. Evaluation of the stress–strain behavior of the cellulose–PAN–Al_2_O_3_ composite separator confirmed its high tensile strength; specifically, 16.15 MPa for the cellulose–PAN separator and 20.83 MPa for the cellulose–PAN–Al_2_O_3_ composite separator (Figure 2d). This result is much higher than conventional GF separators (3.12 MPa) (Zhang et al., 2019). Furthermore, the cellulose–PAN–Al_2_O_3_ composite exhibited a higher puncture strength (2.22 MPa) than the bare separator (0.76 MPa) (Figure 2e). Since tensile and puncture strengths depend on the bonding strength between the alumina particles, they are susceptible to the composition of the alumina and polyvinyl acetate used as a binder in the alumina dip coating process. Thus, we proceeded to the process with a fixed composition.

**Figure 3:**
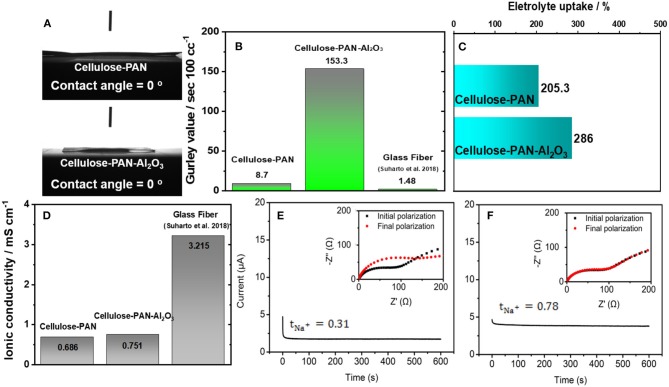
**(A)** Wettability, **(B)** Gurley value, **(C)** Electrolyte uptake, and **(D)** ionic conductivity of electrolyte soaked bare and cellulose–PAN-Al_2_O_3_ composite separator. Sodium transference number of **(E)** bare and **(F)** cellulose–PAN-Al_2_O_3_ composite separator.

The wettability was evaluated by contact angle measurements; the cellulose–PAN and as-prepared cellulose–PAN–Al_2_O_3_ composite separators possessed contact angles of nearly 0° (Figure 3A). The cellulose–PAN separator exhibited excellent wettability, and the Al_2_O_3_ coating did not affect the wettability. To determine the air permeability of the separators, the elapsed time for blowing 100 cc of air was measured; Gurley values of 8.7 s for the cellulose–PAN separator and 153.3 s for the as-prepared cellulose–PAN–Al_2_O_3_ composite separator were obtained (Figure 3B), which are higher than that of the reported value (1.48) for GF separator (Arunkumar et al., 2019). These results imply that the submicron-sized Al_2_O_3_ particles inside and on the surface of the as-prepared cellulose–PAN–Al_2_O_3_ composite separators blocked the air flow owing to the small pore size, as observed in Figure 1b. The electrolyte uptake, ionic conductivity of electrolyte soaked in separator, and Na transference number were evaluated to confirm the effect of the Al_2_O_3_ coating on the affinity for the electrolyte. By comparing the weight of the separator before and after soaking in the electrolyte, it was possible to deduce the electrolyte uptake ability of the separators, as shown in Figure 3C. The weight increases for the cellulose–PAN separator was ~205%, and the absorption ability was increased to 286% for the cellulose–PAN–Al_2_O_3_ composite separator. As seen in Figures 1a–c, the submicron-sized alumina powders were applied on the surface cellulose-PAN layers, and this results the Gurley value higher than cellulose-PAN separator. Since there are many voids between the alumina particles, capillary effect is likely to allow readily penetration of electrolyte, so that contact angle with electrolyte exhibited 0 degree and the electrolyte uptake ratio was comparable to the result of cellulose-PAN separator (Figures 3B,C). Because the electrolyte uptake ability directly affects the ionic conductivity of electrolyte soaked in separator and electrochemical properties, the ionic conductivity and Na^+^ transfer value were subsequently evaluated. The ionic conductivity of electrolyte-soaked separator presented 0.751 mS cm^−1^ (cellulose–PAN–Al_2_O_3_ composite separator) and 0.686 mS cm^−1^ (cellulose–PAN separator) as shown in Figure 3D, which were lower than the ionic conductivity of GF separator (3.215) (Suharto et al., 2018). In addition, the sodium ion transfer number ($t_{\text{Na}}^{+}$) was significantly improved for the cellulose–PAN–Al_2_O_3_ composite separator ($t_{\text{Na}}^{+}$ = 0.78) compared with that of the cellulose–PAN separator ($t_{\text{Na}}^{+}$ = 0.31) (Figures 3E,F). Furthermore, this value is similar with that ($t_{\text{Na}}^{+}$ = 0.79) of GF separator (Arunkumar et al., 2019). Although the presence of alumina in the separator resulted in a higher Gurley value for the cellulose–PAN separator, the added alumina improved the intrinsic properties of electrolyte uptake, ionic conductivity of electrolyte soaked in separator, and sodium-ion transfer number owing to the high surface area provided by the alumina particles (and thereby the interconnected pore structure).

**Figure 4:**
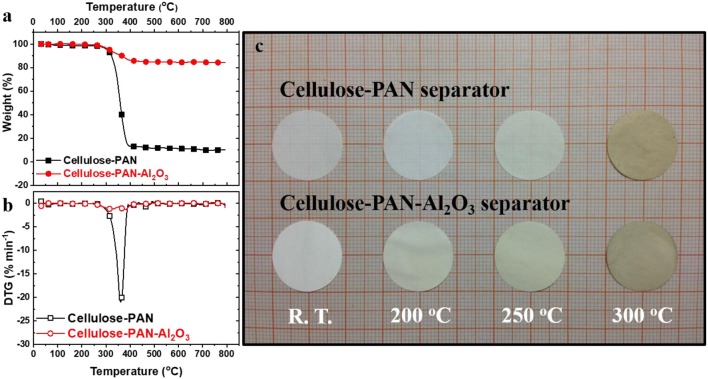
**(a)** TGA curves and **(b)** DTG curves of bare and cellulose–PAN-Al_2_O_3_ composite separator; **(c)** thermal shrinkage rate of bare and cellulose–PAN-Al_2_O_3_ composite separator after heat-treatment in temperature range of 25-300°C.

Thermal stability is another important property for batteries and is directly related to shrinkage of the separator at elevated temperature, which can result in internal short-circuit. The thermal stability must be satisfactory to ensure safety and to extend the operating temperature range of batteries. According to the TG–DTG results, the cellulose–PAN–Al_2_O_3_ composite separator experienced a slight weight loss of ~8% from 250 to 350°C, whereas the cellulose–PAN separator experienced a drastic weight loss of over 90% in the same range (Figure 4a). DTG analysis indicated that the maximum evaporation rate was approximately −20.2% min^−1^ at 359°C for the cellulose–PAN separator; however, the evaporation rate was remarkably reduced to approximately −2.5% min^−1^ at 302°C for the cellulose–PAN–Al_2_O_3_ composite separator (Figure 4b). This preliminary result was confirmed by a shrinkage test, as shown in Figure 4c, which was performed up to 300°C after heat soaking for 30 min. The test results are highly reproducible for both cellulose-PAN and cellulose-PAN-Al_2_O_3_ separators, as we obtained the values in average after tests for 10 times (Figure 4c and Table 1). This improvement is likely associated with the minimized thermal deformation of the cellulose–PAN–Al_2_O_3_ separator supported by the thermally stable alumina particles (Δ*G*_*f*__298K_ = −1582.3 kJ mol^−1^ for formation at 298 K) bound by PVAc, which is stable up to 300°C (Abu-Saied et al., 2012), on the surface and within the separator. Moreover, thermal stability of the cellulose–PAN–Al_2_O_3_ composite separator is much better than that of conventional GF separator, which could not keep the initial shape after heat treatment at 300°C for 1 h (Supplementary Figure 1).

**Table 1:** Thermal shrinking rate.

	**Thermal shrinkage rate**			
**Separator sample**	**R.T. /0.5h**	**200^**°**^C/0.5h**	**250^**°**^C/0.5h**	**300^**°**^C/0.5h**
Cellulose-PAN	0%	0%	0%	4.7%
Cellulose-PAN-Al_2_O_3_	0%	0%	0%	0%

**Figure 5:**
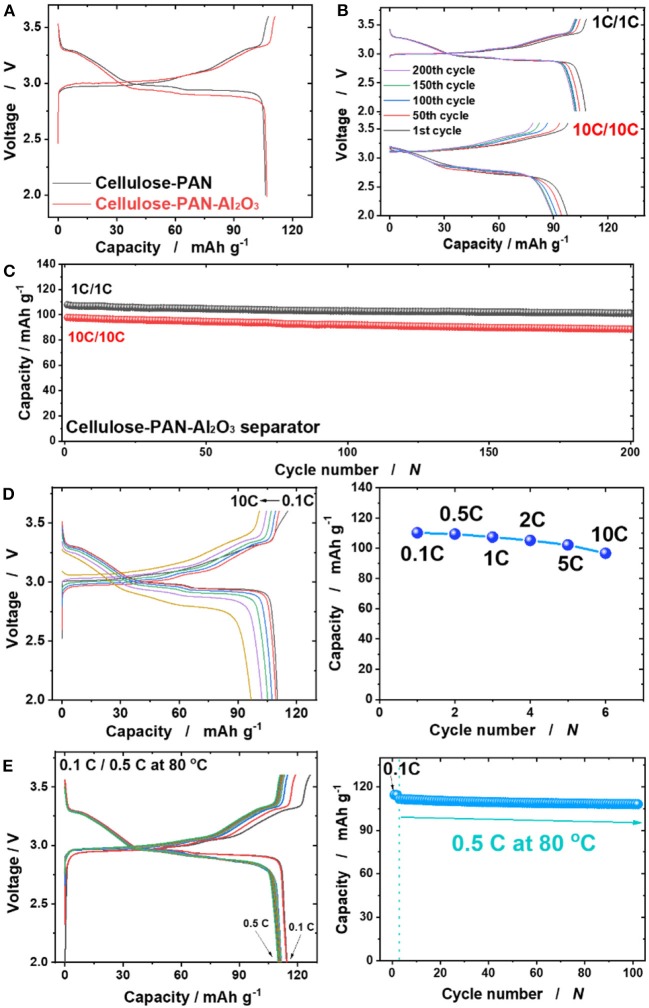
**(A)** First charge and discharge curve of C-NaCrO_2_/Na cells using bare and cellulose–PAN-Al_2_O_3_ composite separator; **(B,C)** cycling performance at rates of 1C and 10C for C-NaCrO_2_/Na cells using cellulose–PAN-Al_2_O_3_ composite separator; **(D)** Rate capability and **(E)** cycling performance (0.5C) at 80°C for C-NaCrO_2_/Na cells using cellulose–PAN-Al_2_O_3_ composite separator.

Figure 5A presents the first charge and discharge curves of C-NaCrO_2_/Na cells using both cellulose–PAN and cellulose–PAN–Al_2_O_3_ composite separators. As a cathode material for SIBs, we thought that various evaluations can be carried out using carbon-coated NaCrO_2_ for separators because of its excellent cycling performance and thermal stability. When cellulose was used as the separator, the cell could not be operated because of the generation of an internal short-circuit resulting from the large pores of the non-woven cellulose (Supplementary Figure 2). Although the operation voltage was slightly lowered on discharge when the cellulose–PAN–Al_2_O_3_ composite separator was adopted, the cell exhibited typical charge and discharge capacities of C-NaCrO_2_, delivering a capacity of ~107 mAh g^−1^ at 1C (110 mA g^−1^) and 97 mAh g^−1^ at 10C (1.1 A g^−1^) with satisfactory cycling performance for 200 cycles, with over 94% retention of the initial capacities (Figures 5B,C). Furthermore, the cells were sustained at intermediate currents (Figure 5D). The cellulose–PAN separator employed in the C-NaCrO_2_ cell could not be cycled over 40 cycles because of deformation of the separator that caused a short-circuit (Supplementary Figure 3), implying the importance of Al_2_O_3_ on the surface and in the cellulose-PAN separator. To confirm the temperature dependence, the operation temperature was increased to 80°C (Figure 5E). The observed capacity was ~112 mAh g^−1^ at 1C with 95% capacity retention for 100 cycles. The slight increase in the capacity resulted from the enhanced ionic diffusivity at elevated temperature (Chandra, 2017). As anticipated from the thermal stability tests that indicated no shrinkage at this temperature (Table 1), operating the cell at 80°C did not induce a short-circuit or abrupt fade in the operation voltage during cycling. Although the cellulose-PAN-Al_2_O_3_ separator developed in this study exhibited lower values of electrolyte uptake (286%) and ionic conductivity (0.75 mS cm^−1^) than the values of GF separator in literature shown above, the cell using the cellulose-PAN-Al_2_O_3_ separator presented the similar electrochemical performance compared to that of cell using glass fiber (Supplementary Figure 4). It thought that Al_2_O_3_ coating layer on the cellulose-PAN separator improve the electrode performance by preventing HF^+^ attacking (Myung et al., 2005; Zhang et al., 2019).

**Figure 6:**
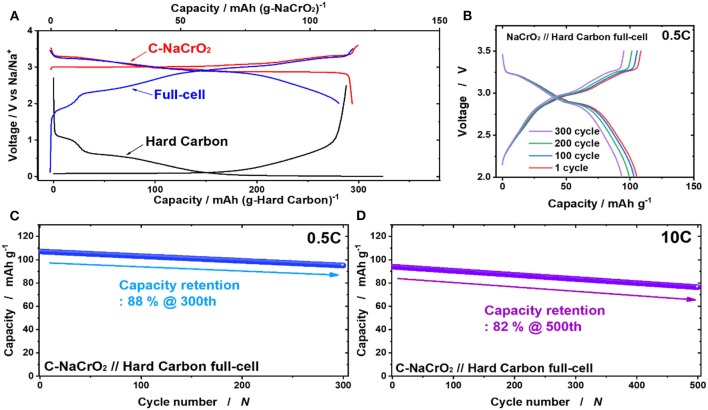
**(A)** First charge and discharge curves of C-NaCrO_2_ half cell (red), hard carbon half cell (black), and C-NaCrO_2_/hard carbon full cell (blue) using cellulose–PAN-Al_2_O_3_ composite separator; **(B,C)** cycling performance of C-NaCrO_2_/hard carbon full cell (blue) using cellulose–PAN-Al_2_O_3_ composite separator. **(D)** Cycling performance at 10 C of C-NaCrO_2_/hard carbon full cell (blue) using cellulose–PAN-Al_2_O_3_ composite separator.

Although the cellulose–PAN–Al_2_O_3_ composite separator was shown to be suitable for cathode application for SIBs, its applicability for full cells is more important to enable its adoption in SIBs. Thus, a pre-sodiated hard carbon anode was paired with the C-NaCrO_2_ cathode, with a N/P capacity ratio of 1.2 (Figure 6A). In the operation range of 2.0–3.5 V at a current of 0.5C at 25°C, the fabricated hard carbon // C-NaCrO_2_ full cell using the cellulose–PAN–Al_2_O_3_ composite separator delivered capacity retention of 88% for 300 cycles (Figures 6B,C). Similar to the half-cell results (Figure 5), the full cell also exhibited satisfactory high-rate performance, resulting capacity retention of 82% at a rate of 10 C after 500 cycles (Figure 6D).

**Figure 7:**
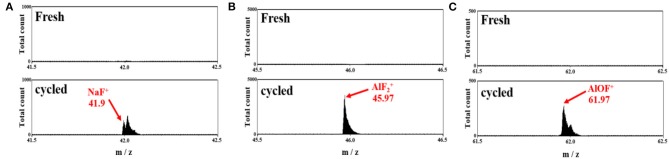
ToF-SIMS data of cellulose–PAN-Al_2_O_3_ composite separator for (top) fresh and (bottom) cycled separator. **(A)** NaF^+^ (m = 41.9), **(B)** ${\text{AlF}}_{2}^{+}$ (m = 45.97), and **(C)** AlOF^+^ (m = 71.97) fragments in a Na cell.

The post-cycled separator was further examined using ToF-SIMS to identify the role of alumina in the cellulose–PAN separator (Figure 7). The NaF^+^ fragment (*m* = 41.98) was detected for the post-cycled cellulose–PAN–Al_2_O_3_ composite separator (Figure 7A), which is indicative of decomposition of the NaPF_6_ salt in the electrolyte. Furthermore, ${\text{AlF}}_{2}^{+}$ (*m* = 45.97, Figure 7B) and AlOF^+^ (*m* = 61.97, Figure 7C) fragments were detected on the surface of the separator. The appearance of these fragments suggests decomposition of the NaPF_6_ salt in the electrochemical environment of Na cells; particularly, the salt can be decomposed in the presence of water molecules that are inevitably contained as an impurity in non-aqueous electrolyte as follows:

(1) $$\begin{array}{l} \text{NaP}{\text{F}}_{6}\to \text{NaF}+\text{P}{\text{F}}_{5} \end{array}$$

(2) $$\begin{array}{l} \text{P}{\text{F}}_{5}+{\text{H}}_{2}\text{O}\to \text{PO}{\text{F}}_{3}+2\text{HF} \end{array}$$

(3) $$\begin{array}{l} 2\text{PO}{\text{F}}_{3}+3\text{N}{\text{a}}_{2}\text{O}\to 6\text{NaF}+{\text{P}}_{2}{\text{O}}_{5}\left(\text{or N}{\text{a}}_{\text{x}}\text{PO}{\text{F}}_{\text{y}}\right) \end{array}$$

**Figure 8:**
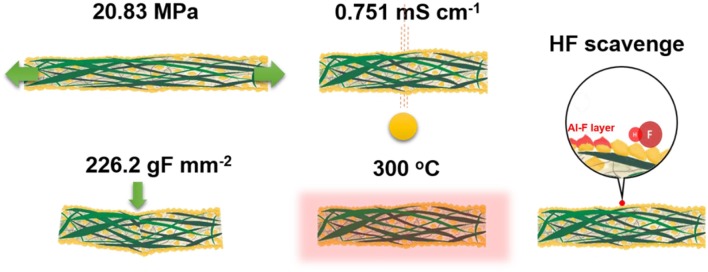
Schematic illustration for the functionalities of a cellulose-PAN-Al_2_O_3_ composite separator.

In the electrolyte, the produced HF faces the loaded amphoteric Al_2_O_3_ particles on the separator, resulting in the following reactions:

(4) $$\begin{array}{l} \text{A}{\text{l}}_{2}{\text{O}}_{3}+2\text{HF}\to \text{A}{\text{l}}_{2}{\text{O}}_{2}{\text{F}}_{2}+{\text{H}}_{2}\text{O} \end{array}$$

(5) $$\begin{array}{l} \text{A}{\text{l}}_{2}{\text{O}}_{2}{\text{F}}_{2}+2\text{HF}\to \text{A}{\text{l}}_{2}\text{O}{\text{F}}_{4}+{\text{H}}_{2}\text{O} \end{array}$$

(6) $$\begin{array}{l} \text{A}{\text{l}}_{2}\text{O}{\text{F}}_{4}+2\text{HF}\to 2\text{Al}{\text{F}}_{3}+{\text{H}}_{2}\text{O} \end{array}$$

leading to fluorination of Al_2_O_3_ toward AlF_3_ via the above scavenging process. Thus, it is beneficial to use alumina with a cellulose–PAN separator to improve the electrode performance. Furthermore, our previous research reported that AlF_3_ layer formed by scavenging HF plays an important role to act as a passive layer at high voltage in Na cells (Konarov et al., 2019). Bugga et al. also presented AlF_3_ coated separator, demonstrating that the AlF_3_ layer also protected separator surface, preventing short-circuit by dendrites and significant impact than Al_2_O_3_ coating on the cycling stability (Bugga et al., 2018; Sun et al., 2018). For the above reasons, the cell using cellulose-PAN-Al_2_O_3_ separator presented the good electrochemical performance. The functionality of Al_2_O_3_ on the cellulose–PAN separator is schematically illustrated in Figure 8.

## Conclusion

This is the first report on the feasibility of cellulose–PAN–Al_2_O_3_ composites as separators for SIBs. The electrochemical, thermal, and mechanical performance of the cellulose–PAN separator was extraordinary improved with the addition of alumina. This new type of cellulose–PAN-Al_2_O_3_ composite separator presented multi-functional abilities with excellent thermal stability at 300°C, a large sodium-ion transport number ($t_{\text{Na}}^{+}$ = 0.78), improved ionic conductivity of electrolyte soaked in separator (0.751 mS cm^−1^), and HF scavenging ability during electrochemical cycling. Half-cells using the separator delivered a high capacity of 107 mAh g^−1^ at a rate of 1C with acceptable capacity retention at both 25° and 80°C. Moreover, a full cell using the cellulose–PAN–Al_2_O_3_ composite separator also exhibited excellent cycling stability of 88% for 300 cycles. Our findings provide insight for the development of alternatives to glass fiber separators for SIBs, which will help enable the commercial availability of SIBs in the near future.

## Data Availability Statement

All datasets generated for this study are included in the article/Supplementary Material.

## Author Contributions

JJ, C-HJ, LS, ZW, SY, and S-TM designed the whole experiment, and wrote the paper. ZQ performed the experiment for separator. HY conducted ToF-SIMS measurement.

### Conflict of Interest

The authors declare that the research was conducted in the absence of any commercial or financial relationships that could be construed as a potential conflict of interest.

## References

[B1] Åvall G. Mindemark J. Brandell D. Johansson P. (2018). Sodium-Ion battery electrolytes: modeling and simulations. Adv. Energy Mater. 8:1703036 10.1002/aenm.201703036

[B2] Abu-Saied M. A. Khalil K. A. Al-Deyab S. S. (2012). Preparation and characterization of poly vinyl acetate nanofiber doping copper metal. Int. J. Electrochem. Sci. 7, 2019–2027. Available online at: http://www.electrochemsci.org/list12.htm#issue3

[B3] Amatucci G. G. Tarascon J. M. Klein L. C. (1996). CoO_2_, the end member of the Li_x_CoO_2_ solid solution. J. Electrochem. Soc. 143, 1114–1123, 10.1149/1.1836594

[B4] Arunkumar R. Ajay P. V. K. S. Ramaprabhu S. (2019). Barium titanate-based porous ceramic flexible membrane as a separator for room-temperature sodium-ion battery. ACS Appl. Mater. Interfaces 11, 3889–3896. 10.1021/acsami.8b17887 30605300

[B5] Bugga R. Jones J.-P. Jones S. C. Krause F. C. Pasalic J. Ganapathi D. S. (2018). New separators in lithium/sulfur cells with high-capacity cathodes. J. Electrochem. Soc. 165, A6021–A6028. 10.1149/2.0051801jes

[B6] Chandra A. (2017). Temperature dependent ionic conductivity and cell performance studies of hot-pressed nanocomposite polymer electrolytes. Compos. Commun. 4, 33–36. 10.1016/j.coco.2017.04.001

[B7] Chen W. Zhang L. Liu C. Feng X. Zhang J. Guan L.. (2018). Electrospun flexible cellulose acetate-based separators for sodium-ion batteries with ultralong cycle stability and excellent wettability: the role of interface chemical groups. ACS Appl. Mater. Inter. 10, 23883–23890. 10.1021/acsami.8b06706 29920205

[B8] Chiapponea A. Naira J. R. Gerbaldib C. Jabbourc L. Bongiovannia R. Zenod E. (2011). Microfibrillated cellulose as reinforcement for Li-Ion battery polymer electrolytes with excellent mechanical stability. J. Power Sources. 196, 10280–10288. 10.1016/j.jpowsour.2011.07.015

[B9] Choi J. U. Jo J. H. Jo C.-H. Cho M. K. Park Y. J. Jin Y. (2019). Impact of Na_2_MoO_4_ nanolayers autogenously formed on tunnel-type Na_0.44_MnO_2_. J. Mater. Chem. 7, 13522–13530. 10.1039/C9TA03844B

[B10] Choi J. U. Park Y. J. Jo J. H. Kuo L. Y. Kaghazchi P. Myung S.-T. (2018). Unraveling the role of earth-abundant fe in the suppression of jahn–teller distortion of P′2-Type Na_2/3_MnO_2_: experimental and theoretical studies. ACS Appl. Mater. Inter. 10, 40978–40984. 10.1021/acsami.8b16522 30431251

[B11] Chun S. J. Lee S. Y. Doh G. H. Lee S. Kim J. H. (2011). Preparation of ultrastrength nanopapers using cellulose nanofibrils. J. Ind. Eng. Chem. 17, 521–526. 10.1016/j.jiec.2010.10.022

[B12] Guignard M. Didier C. Darriet J. Bordet P. Elkaïm E. Delmas C. (2013). P2-Na_x_VO_2_ system as electrodes for batteries and electron-correlated materials. Nat. Mater. 12, 74–80. 10.1038/nmat3478 23142842

[B13] Hwang J.-Y. Myung S.-T. Sun Y.-K. (2017). Sodium-Ion batteries: present and future. Chem. Soc. Rev. 46, 3529–3614. 10.1039/c6cs00776g 28349134

[B14] Jeng Y.-B. Kim D.-W. (2004). Effect of thickness of coating layer on polymer-coated separator on cycling performance of lithium-ion polymer cells. J. Power Sources 128, 256–262. 10.1016/j.jpowsour.2003.09.073

[B15] Jo C. H. Cho D. H. Noh H. J. Yashiro H. Sun Y. K. Myung S.-T. (2015). An effective method to reduce residual lithium compounds on Ni-rich Li[Ni_0.6_Co_0.2_Mn_0.2_]O_2_ active material using a phosphoric acid derived Li_3_PO_4_ nanolayer. Nano Res. 8, 1464–1479. 10.1007/s12274-014-0631-8

[B16] Jo C. H. Jo J. H. Yashiro H. Kim S. J. Sun Y.-K. Myung S.-T. (2018a). Bioinspired surface layer for the cathode material of high-energy-density sodium-ion batteries. Adv. Ener. Mater. 8:1702942 10.1002/aenm.201702942

[B17] Jo C. H. Yashiro H. Shuai Y. Shi L. Myung S.-T. (2018b). Conversion chemistry of cobalt oxalate for sodium storage. ACS Appl. Mater. Inter. 10, 40523–20530. 10.1021/acsami.8b13641 30371051

[B18] Jo J. H. Choi J. U. Cho M. K. Aniskevich Y. Kim H. Ragoisha G. (2019). Hollandite-Type VO_1.75_(OH)_0.5_: effective sodium storage for high-performance sodium-ion batteries. Adv. Energy Mater. 9:1900603 10.1002/aenm.201900603

[B19] Jo J. H. Choi J. U. Konrov A. Yashiro H. Yuan S. Shi L. (2018). Sodium-ion batteries: building effective layered cathode materials with long-term cycling by modifying the surface via sodium phosphate. Adv. Funct. Mater. 28:1705968 10.1002/adfm.201705968

[B20] Kim D.-W. Ko J.-M. Chun J.-H. Kim S.-H. Park J.-K. (2001). Electrochemical performances of lithium-ion cells prepared with polyethylene oxide-coated separators. Electrochem. Commun. 3, 535–538. 10.1016/S1388-2481(01)00214-4

[B21] Kim H. Son S. Choi W. I. Park G. O. Kim Y. Kim H. (2018). Direct observation of pseudocapacitive sodium storage behavior in molybdenum dioxide anodes, J. Power Sources. 398, 113–123. 10.1016/j.jpowsour.2018.07.010

[B22] Kim J. S. Seo D. H. Kim H. S. Park I. C. Yoo J.-K. Jung S.-K. (2015). Unexpected discovery of low-cost maricite NaFePO_4_ as a high-performance electrode for Na-Ion batteries. Energy. Environ. Sci. 8, 540–545. 10.1039/C4EE03215B

[B23] Kim J. Y. Kim S. K. Lee S.-J. Lee S. Y. Lee H. M. Ahn S. (2004). Preparation of micro-porous gel polymer for lithium ion polymer battery. Electrochim. Acta 50, 363–366. 10.1016/j.electacta.2004.01.131

[B24] Kim J. Y. Lee Y. Lim D.-Y. (2009). Plasma-modified polyethylene membrane as a separator for lithium-ion polymer battery. Electrochim. Acta 54, 3714–3719. 10.1016/j.electacta.2009.01.055

[B25] Kim K. J. Kwon H. K. Park M.-S. Yim T. Yu J.-S. Kim Y.-J. (2014). Ceramic composite separators coated with moisturized ZrO_2_ nanoparticles for improving the electrochemical performance and thermal stability of lithium ion batteries. Phys. Chem. Chem. Phys. 16, 9337–9343. 10.1039/C4CP00624K 24715040

[B26] Komaba S. Ishikawa T. Yabuuchi N. Murata W. Ito A. Ohsawa Y. (2011). Fluorinated ethylene carbonate as electrolyte additive for rechargeable na batteries. ACS Appl. Mater. Interfaces, 3, 4165–4168. 10.1021/am200973k 22026720

[B27] Konarov A. Jo J. H. Choi J. U. Bakenov Z. Yashiro H. Kim J. (2018). Exceptionally highly stable cycling performance and facile oxygen-redox of manganese-based cathode materials for rechargeable sodium batteries. Nano Envergy 59, 197–206. 10.1016/j.nanoen.2019.02.042

[B28] Konarov A. Kim H. J. Yashiro H. Myung S.-T. (2019). Passivation of aluminum current collectors in non-aqueous carbonate solutions containing sodium or potassium hexafluorophosphate salts. J. Mater. Chem. 7, 13012–13018. 10.1039/C9TA03911B

[B29] Lee Y. Yoo J.-K. Jo J. H. Park H. Jo C.-H. Ko W.. (2019). The conversion chemistry for high-energy cathodes of rechargeable sodium batteries. ACS Nano, 13, 11707–11716. 10.1021/acsnano.9b05635 31600049

[B30] Mishra S. K. Ceder G. (1999). Structural stability of lithium manganese oxides. Phys. Rev. 59:6120 10.1103/PhysRevB.59.6120

[B31] Mizushima K. Jones P. C. Wiseman P. J. Goodenough J. B. (1980). Li_x_CoO_2_ (0 < x < -1): A new cathode material for batteries of high energy density. Mater. Res. Bull. 15, 783–789. 10.1016/0025-5408(80)90012-4

[B32] Myung S.-T. Izumi K. Komaba S. Sun Y.-K. Yashiro H. Kumagai N. (2005). role of alumina coating on Li–Ni–Co–Mn–O particles as positive electrode material for lithium-ion batteries. Chem. Mater. 17, 3695–3704. 10.1021/cm050566s

[B33] Nithya C. Gopukumar S. (2015). Sodium ion batteries: a newer electrochemical storage. Adv. Rev. 4, 253–278. 10.1002/wene.136

[B34] Pan H. Hu Y. S. Chen L. (2013). Room-temperature stationary sodium-ion batteries for large-scale electric energy storage. Energy Environ. Sci. 6, 2338–2360. 10.1039/c3ee40847g

[B35] Park J. S. Jo J. H. Yashiro H. Kim S. S. Kim S. -J. Sun Y.-K. (2017). Synthesis and electrochemical reaction of tin oxalate-reduced graphene oxide composite anode for rechargeable lithium batteries. ACS Appl. Mater. Inter. 31, 25941–25951. 10.1021/acsami.7b03325 28718628

[B36] Sato T. Sato K. Zhao W. Kajiya Y. Yabuuchi N. (2018). Metastable and nanosize cation-disordered rocksalt-type oxides: revisit of stoichiometric LiMnO_2_ and NaMnO_2_. J. Mater. Chem. 6, 13943–13951. 10.1039/C8TA03667E

[B37] Siró I. Plackett D. (2010). Microfibrillated cellulose and new nanocomposite materials: a review. Cellulose 17, 459–494. 10.1007/s10570-010-9405-y

[B38] Suharto Y. Lee Y. Yu J.-S. Choi W. Kim K. J. (2018). Microporous ceramic coated separators with superior wettability for enhancing the electrochemical performance of sodium-ion batteries. J. Power Sources 376, 184–190. 10.1016/j.jpowsour.2017.11.083

[B39] Sun H.-H. Hwang J.-Y. Yoon C. S. Heller A. Mullins C. B. (2018). Capacity degradation mechanism and cycling stability enhancement of AlF3-coated nanorod gradient Na[Ni_0.65_Co_0.08_Mn_0.27_]O_2_ cathode for sodium-ion batteries. ACS Nano, 12, 12912–12922. 10.1021/acsnano.8b08266 30475595

[B40] Sun Y. K. Myung S. T. Park B. C. Prakash J. Belharouak I. Amine K. (2009). High-energy cathode material for long-life and safe lithium batteries. Nat. Mater. 8, 320–324. 10.1038/nmat2418 19305398

[B41] Vaalma C. Buchholz D. Weil M. Passerini S. A. (2018). Cost and resource analysis of sodium-ion batteries. Nat. Rev. Mater. 3:18013 10.1038/natrevmats.2018.13

[B42] Vassilaras P. Ma X. Li X. Ceder G. (2013). Electrochemical properties of monoclinic NaNiO_2_. J. Electrochem. Soc. 160, A207–A211. 10.1149/2.023302jes

[B43] Wang J. Wang Y. Seo D.-H. Shi T. Chen S. Tian Y. (2020). A high-energy NASICON-type cathode material for Na-Ion batteries. Adv. Energy Mater. 1903968. 10.1002/aenm.201903968

[B44] Winter M. Besenhard J. O. Spahr M. E. Novak P. (1998). Insertion electrode materials for rechargeable lithium batteries. Adv. Mater. 10, 725–763. 10.1002/(sici)1521-4095(199807)10:10<725::aid-adma725>3.0.co;2-z

[B45] Xu K. (2004). Nonaqueous liquid electrolytes for lithium-based rechargeable batteries. Chem. Rev. 104, 4303–4418. 10.1021/cr030203g 15669157

[B46] Yabuuchi N. Kajiyama M. Iwatate J. Nishikawa H. Hitomi S. Okuyama R. (2012). P2-type Na_x_[Fe_1/2_Mn_1/2_]O_2_ made from earth-abundant elements for rechargeable na batteries. Nat. Mater. 11, 512–517. 10.1038/nmat3309 22543301

[B47] Yabuuchi N. Yano M. Yoshida H. Kuze S. Komaba S. (2013). Synthesis and electrode performance of O_3_-Type NaFeO_2_-NaNi_1/2_Mn_1/2_O_2_ solid solution for rechargeable sodium batteries. J. Electrochem. Soc. 160, A3131–A3137. 10.1149/2.018305jes

[B48] Yu C. Park J.-S. Jung H.-G. Chung K.-Y. Aurbach D. Sun Y.-K. (2015). NaCrO_2_ cathode for high-rate sodium-ion batteries. Ener. Environ. Sci. 8, 2019–2026. 10.1039/C5EE00695C

[B49] Zhang Y. Liu L. Jamila S. Xie J. Liu W. Xia J. (2019). Al_2_O_3_ coated Na_0.44_MnO_2_ as high-voltage cathode for sodium ion batteries. Appl. Surf. Sci. 494, 1156–1165. 10.1016/j.apsusc.2019.07.247

[B50] Zhu J. Yanilmaz M. Fu K. Chen C. Lu Y. Ge Y. (2016). Understanding glass fiber membrane used as a novel separator for lithium-sulfur batteries. J. Membr. Sci. 504, 89–96. 10.1016/j.memsci.2016.01.020

