# Supplementary material for: Nature-Derived Cellulose-Based Composite Separator for Sodium-Ion Batteries
Source: Front Chem. 2020 Mar 10;8:153. doi: 10.3389/fchem.2020.00153 (PMC7076124; doi:10.3389/fchem.2020.00153)
Supplement: Supplementary file 1 [file Data_Sheet_1.pdf]

Supplementary Material

Nature-derived Cellulose-based Composite Separator for Sodium-ion Batteries

Jae Hyeon Jo[1,z], Chang-Heum Jo[1,z], Zhengfu Qiu[2], Hitoshi Yashiro[3], Liyi Shi[2], Zhuyi Wang[2], Shuai Yuan[2*] and Seung-Taek Myung[1*]

[1]*Department* of Nanotechnology and Advanced Materials Engineering & Sejong Battery Institute, Sejong University, Seoul, 05006 South Korea

[2]Research Centre of Nanoscience and Nanotechnology, Shanghai University, Shanghai 200444, China

[3]Department of Chemistry and Bioengineering, Iwate University, Ueda 4-3-5, Morioka, Iwate 020-8551, Japan

*** Correspondence:**
Shuai Yuan
s.yuan@shu.edu.cn
Seung-Taek Myung
smyung@sejong.ac.kr

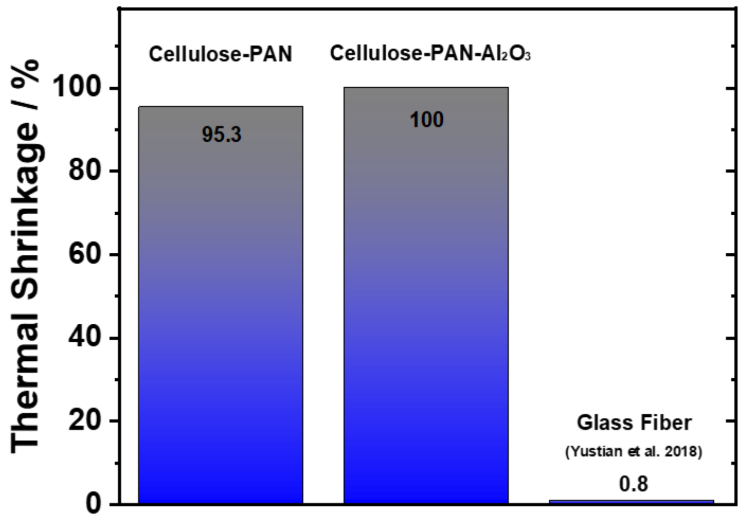

Supplementary Figure 1. Thermal shrinkage rate for Cellulose-PAN, Cellulose-PAN-Al_2O_3 and Glass Fiber (300 ^oC for 0.5h)

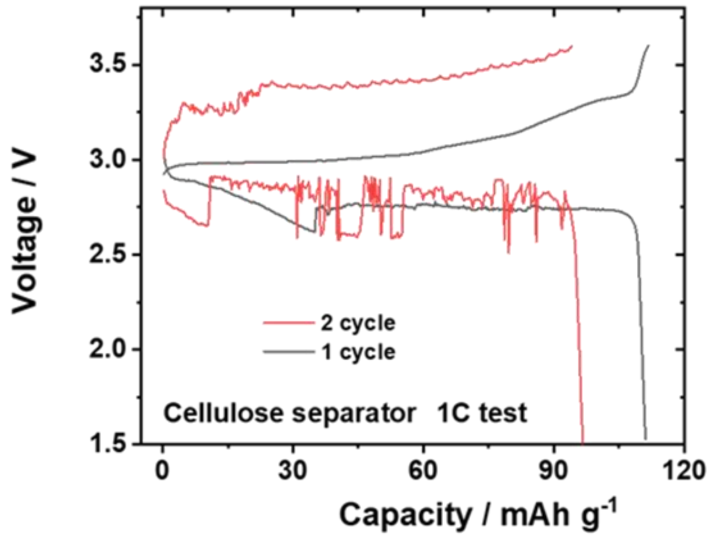

Supplementary Figure 2. Initial charge and discharge curves of C-$NaCrO_2$ cell using cellulose separator.

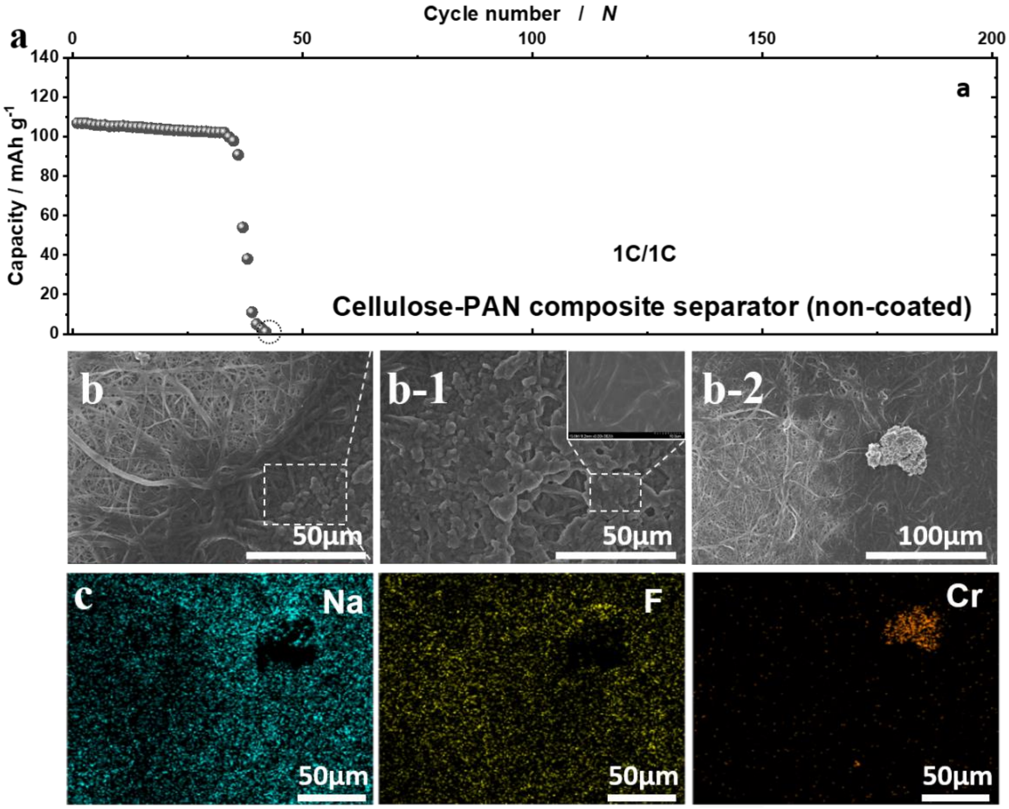

Supplementary Figure 3. (a) Cycling performance of C-$NaCrO_2$ using uncoated cellulose–PAN composite separator. (b) SEM images of surface of cycled uncoated cellulose–PAN composite separator and (c) EDX mapping images for (b-2).

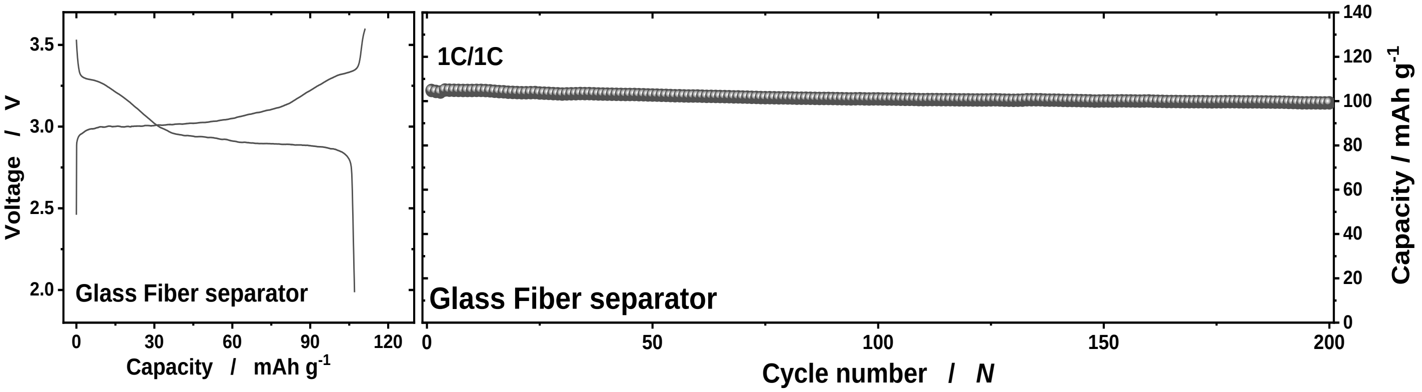

Supplementary Figure 4. (a) charge and discharge curves of C-$NaCrO_2$/Na cells using glass fiber separator; (b) cycling performance at rates of 1C.
